# A novel logistic regression model combining semi-supervised learning and active learning for disease classification

**DOI:** 10.1038/s41598-018-31395-5

**Published:** 2018-08-29

**Authors:** Hua Chai, Yong Liang, Sai Wang, Hai-wei Shen

**Affiliations:** Faculty of Information Technology & State Key Laboratory of Quality Research in Chinese Medicines, Macau University of Science and Technology, Avenida Wai Long, Taipa Macau, 999078 China

## Abstract

Traditional supervised learning classifier needs a lot of labeled samples to achieve good performance, however in many biological datasets there is only a small size of labeled samples and the remaining samples are unlabeled. Labeling these unlabeled samples manually is difficult or expensive. Technologies such as active learning and semi-supervised learning have been proposed to utilize the unlabeled samples for improving the model performance. However in active learning the model suffers from being short-sighted or biased and some manual workload is still needed. The semi-supervised learning methods are easy to be affected by the noisy samples. In this paper we propose a novel logistic regression model based on complementarity of active learning and semi-supervised learning, for utilizing the unlabeled samples with least cost to improve the disease classification accuracy. In addition to that, an update pseudo-labeled samples mechanism is designed to reduce the false pseudo-labeled samples. The experiment results show that this new model can achieve better performances compared the widely used semi-supervised learning and active learning methods in disease classification and gene selection.

## Introduction

Identifying disease related genes and classifying the disease type using gene expression data is a very hot topic in machine learning. Many different models such as logistic regression model^1^ and support vector machines (SVM)^2^ have been applied in this area. However these supervised learning methods need a lot of labeled samples to achieve satisfactory results. Nevertheless in many biological datasets there is only a small size labeled data and remaining samples are unlabeled. Labeling these unlabeled samples manually is difficult or expensive; hence many unlabeled samples are left in the dataset. On the other hand, the proportion of small size labeled samples may not represent the real data distribution, which makes the classifier difficult to get the expected accuracy. Trying to improve the classification performance, many incrementally learning technologies such as semi-supervised learning (SSL)^3^ and active learning (AL)^4^ have been designed which utilize the unlabeled samples.

AL tries to train an accurate prediction model with minimum cost of labeling the unlabeled samples manually. It selects most uncertain or informative unlabeled samples and annotates them by human experts. These labeled samples are included to the training dataset to improve the model performance. Uncertainty sampling^5^ is the most popular AL strategy in practice because it does not require significant overhead to use. However one problem is that using uncertainty sampling may make the model to be short-sighted or biased^6^. What is more, though AL reduces the manpower work, manually labeling the selected samples by AL in biological experiments still cost much.

In another way, SSL uses unlabeled data together with labeled data in the training process without any manual labeling. Many different SSL methods have been designed in machine learning including transductive support vector machines^7^, graph-based methods^8^, co-training^9^, self-training^10^ and so on. However^11^ pointed out that the pseudo-labeled samples are annotated based on the labeled samples in the dataset, and they are easy to be affected by the high noisy samples. That is why SSL may not achieve satisfactory accuracy in some places.

Many researchers found the complementarity between AL and SSL. Song combined the AL and SSL to extract protein interaction sentences^12^, the most informative samples which were selected by AL-SVM were annotated by experts and then the classifier was retrained using SSL technology by the new dataset^13^. used a SSL technology to help AL select the query points more efficiently and further reducing the workload of manual classification. In^14^ a SVM classifier was proposed to manually label the most uncertain samples and at the same time the other unlabeled samples were labeled by SSL, thus a faster convergence result was gained. The recent study^15^ proposed by Lin designed a new active self-paced learning mechanism which combines the AL and SSL for face recognition.

However, most attention of the methods combing SSL and AL are paid to the SVM model. The logistic regression model which widely used for disease classification is seldom mentioned. And also in these existing methods, the most informative samples selected by AL are manually annotated, this work maybe very expensive or time consuming in disease classification. Hence we design a new logistic regression model combining AL and SSL which meets the following requirements:

*The new model should be easily understood and applied. Our method should not require significant engineering overhead to use*.

In this new logistic regression model, we use uncertainty sampling to select the most informative samples in AL. Uncertainty sampling is fairly easily generalized to probabilistic structure prediction models. For logistic regression model, the sample probability closed to the decision boundary (probability ≈ 0.5) will suffice. In the new logistic regression model, self-training is used as a complement to AL. Self-training is one of the popular technologies used in SSL because of its fast speed and simplicity, and this method is a good way to solve the short-sighted problem in AL. In self-training the classifier is first trained by using the small size labeled samples, and then the obtained classifier will be used to label the high confidence samples in the unlabeled samples pool. These selected samples will be included into the training set and the classifier will be retrained. The cycle repeats until all the unlabeled samples have been used. In the logistic regression model, the samples which the probability closed to 0 or 1 can be seen as the high confidence samples. In our model, uncertainty sampling is used for avoiding the classifier being misled by high noisy samples, and self-training can avoid the model to be short-sighted or biased because of the high confidence samples’ compactness and consistency in the feature space^15^. By the complementarity of uncertainty sampling and self-training, it is easy to build a select-retrain circulation mechanism based on the samples’ probabilities estimated by the logistic classifier.

*The new model can achieve a satisfactory accuracy while labeling the samples automatically without manual labeling*.

Sometimes labeling the disease samples manually is difficult, expensive or time consuming. In our model the uncertain samples selected by AL are labeled by the last classifier automatically, it significantly reduces the burden of manual labeling. However how to ensure the correctness of these uncertain samples? The most uncertain samples mean the false pseudo-labeled samples are easy to be generated. On the other hand the most uncertain samples can be seen as the most informative samples in the logistic model, and the misclassified samples will degenerate the model performance obviously. Considering these samples are not removed or corrected in the standard AL and SSL methods, we design an update mechanism for the pseudo-labeled samples which makes the misclassified samples have chances to be corrected based on the new classifiers which generated in later training interactions.

## Method

### Logistic regression model

Supposing the biological dataset has *n* samples, which includes *n*_*1*_ labeled samples and *n*_*2*_ unlabeled samples, *n* = *n*_*1*_ + *n*_*2*_. And this dataset contains *p* genes. $\beta \,(\beta ={\beta }_{0}+{\beta }_{1}+{\beta }_{2}\ldots +{\beta }_{p})$ represents the coefficients between the disease type *Y* and gene expression *X*. ${({y}_{i},{c}_{i},{x}_{i})}_{i}^{n}$ represents the individual sample, where *y*_*i*_ is disease type, ${x}_{i}=({x}_{i1},{x}_{i2},\ldots {x}_{ip})$ represents the gene expression data, *c*_*i*_ represents the sample is labeled or unlabeled. The basic logistic regression model can be expressed as:

1 $$P({y}_{i}=1|{x}_{i})=\frac{\exp ({x}_{i}\beta )}{1+\exp ({x}_{i}\beta )}\,$$

The log-likelihood of the logistic regression method can be expressed as:

2 $$l(\beta )=-\,{\sum }_{i=1}^{n}\{{y}_{i}log[\frac{\exp ({x}_{i}\beta )}{1+\exp ({x}_{i}\beta )}]+(1-{y}_{i})log[\frac{\exp ({x}_{i}\beta )}{1+\exp ({x}_{i}\beta )}]\}$$

Trying to identify disease related genes in the gene expression data, L1-norm regularization (Lasso) is added in the model:

3 $${\rm{\min }}\,{\sum }_{i=1}^{n1}l({x}_{i}^{T},{y}_{i},\beta )+\lambda P(\beta )$$

Where $P({\beta }_{j})\,\,$is the L1-norm regularization part and *λ* is the tuning parameter.

### Uncertainty sampling

In the active learning part of our method, we use uncertainty sampling to select samples in the unlabeled dataset. In the logistic regression model the sample which probability close to the decision boundary (probability ≈ 0.5) can be seen as the most uncertain sample in AL. Hence an AL logistic regression model can be expressed as:

4 $${\rm{\min }}\,{\sum }_{i=1}^{n1}l({x}_{i}^{T},{y}_{i},\beta )+{\sum }_{j=1}^{n2}{v}_{j}l({x}_{j}^{T},{y}_{i},\beta )+{f}_{AL}({v}_{j},\alpha )+\lambda P(\beta )$$

where *v* is the weight parameter of the unlabeled samples, and the ${f}_{AL}(v,\alpha )$ represents the selection function which can be used to generate the *v*, *a* is the control parameter. The selected unlabeled samples will be labeled manually and then included into the training dataset. The ${f}_{AL}(v,\alpha )$ can be expressed as following:

5 $${v}_{j}=\{\begin{array}{cc}1 & 0.5-\alpha  < \,\,l({x}_{j}^{T},{y}_{i},\beta ) < \,0.5+\alpha \\ 0 & \,\,\,\,\,\,\,\,\,\,\,\,else\,\end{array}$$

### Self-training

In the logistic regression model the sample probability closest to 0 or 1 can be seen as the high confidence sample. It is easy to find that the difference between the self-training and uncertainty sampling is that the selection criteria of identifying the used unlabeled samples. Hence the self-training logistic regression model is shown as:

6 $${\rm{\min }}\,{\sum }_{i=1}^{n1}l({x}_{i}^{T},{y}_{i},\beta )+{\sum }_{j=1}^{n2}{w}_{j}\,l({x}_{j}^{T},{y}_{i},\beta )+{f}_{SSL}({w}_{j},\gamma )+\lambda P(\beta )$$

where *w* is the weight parameter of the unlabeled samples, the ${f}_{SSL}(w,\gamma )$ represents the selection function of self-training and *γ* is the control parameter. The ${f}_{SSL}(w,\gamma )$ is shown as:

7 $${w}_{j}=\{\begin{array}{cc}0 & \gamma  < \,l({x}_{j}^{T},{y}_{i},\beta ) < \,1-\gamma \\ 1 & \,\,\,\,\,\,\,else\end{array}$$

### The logistic regression model combining semi-supervised learning and active learning

In this paper we propose a novel logistic regression model combining SSL and AL with an update mechanism. The high confidence unlabeled samples selected by self-training can avoid the classifier to be short-sighted. The low confidence samples selected by uncertainty sampling prevent the classifier to be misled by high noisy samples which are offered by self-training. The model can be expressed as:

8 $${\rm{\min }}\,{\sum }_{i=1}^{n1}wl({x}_{i}^{T},{y}_{i},\beta )+{\sum }_{j=1}^{n2}({w}_{j}\oplus {v}_{j})l({x}_{j}^{T},{y}_{i},\beta )+{f}_{AL}({v}_{j},\alpha )+{f}_{SSL}({w}_{j},\gamma )+\lambda P(\beta )$$

where *w* is the weight parameter of the unlabeled samples given by SSL, and the *v* is the weight parameter of the unlabeled samples obtained by AL.

Different from the ordinary AL methods, the unlabeled samples selected in our model are labeled by the learned classifier automatically. Considering the uncertainty of classified samples, the misclassified samples should have the chances to be revised in latter training iterations. The update mechanism is described below:

- If the sample is selected by SSL and the label has been changed by the classifier, this sample will be returned to the unlabeled sample pool and wait to be selected again.
- If the sample is selected by AL and the label has been changed, we revise the label of this sample and it will be put into the training dataset directly.

**Figure 1:**
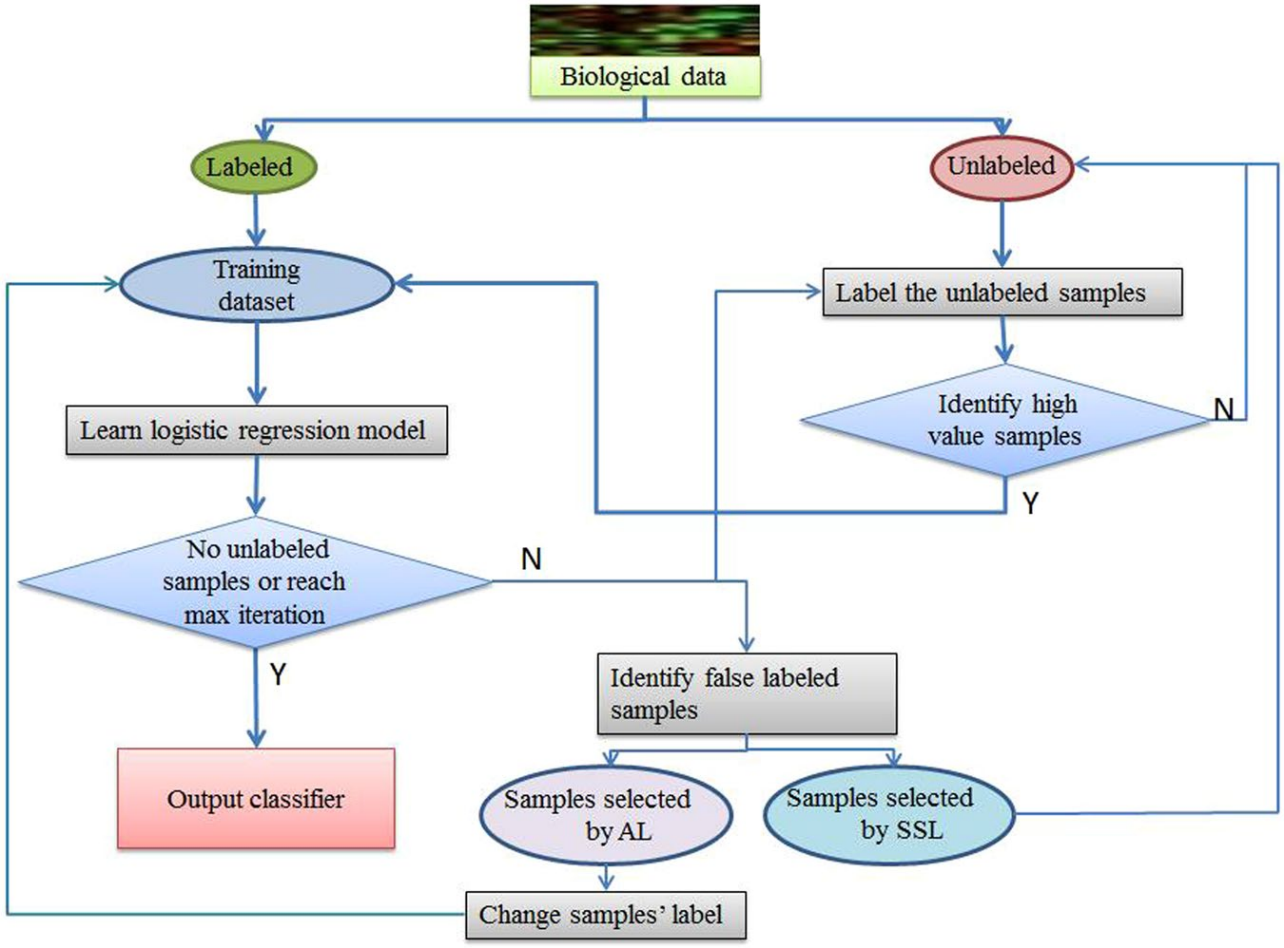
The work flow of proposed logistic regression model combining SSL and AL.

The work flow of our proposed logistic regression model is show in Fig. 1:

- Step 1: Firstly the labeled data will be used to learn an initial logistic regression model.
- Step 2: The logistic regression model will be used to label the unlabeled samples and the high value samples which are selected by SSL or AL will be included into the training dataset.
- Step 3: Update the logistic regression model using the new training dataset.
- Step 4: Identify the false pseudo-labeled samples. If they are selected by SSL, return them to the unlabeled sample pool. Otherwise, change their labels and put them into the training dataset directly.
- Step 5: The cycle will continue until all the unlabeled samples have been labeled or the run time exceeds the maximum number of iteration.

The algorithm of our proposed logistic regression model combining SSL and AL is given in below:

**Algorithm 1:**
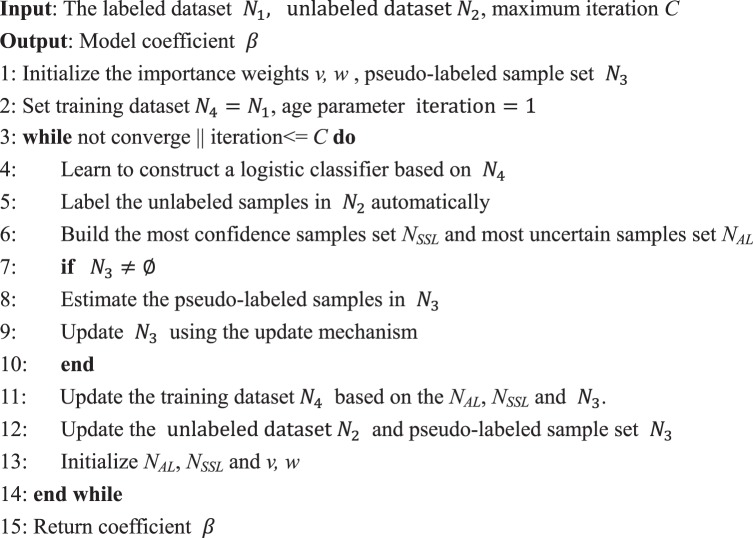
The algorithm of the semi-supervised logistic regression model.

The maximum iteration *C* is computed based on the step size *SZ* which is the selection range for identifying high value samples based on the pseudo-labeled samples’ probabilities. The *prob*_*i*_ is defined as the probability of the *i*_*th*_ pseudo-labeled sample which is estimated by the logistic model. Here we give an example to discuss the convergence of this model: if the *SZ* is set 0.2, the *C* is 5 (*SZ* *C = 1). In first iteration only the pseudo-labeled samples meeting the following conditions will be used: 0 < *prob*_*i*_ < 0.05 and 0.95 < *prob*_*i*_ < 1 (selected by SSL) & 0.45 < *prob*_*i*_ < 0.55 (selected by AL), here the initial probability range is 0.2; in the second iteration the range will be increased to 0 < *prob*_*i*_ < 0.1 and 0.9 < *prob*_*i*_ < 1 (selected by SSL) & 0.4 < *prob*_*i*_ < 0.6 (selected by AL), the probability range is increased to 0.4, *SZ* = 0.2 means in every iteration the range of probability will increase by 0.2. And while *C* = 5, the probability range is increased to 1, it means all the pseudo-labeled samples will be used. The commonly *C* is set 10 (*SZ* = 0.1) or 20 (*SZ* = 0.05). Sometimes before the iteration reaches the maximum iteration *C*, all the pseudo-labeled samples have been selected, especially while the *SZ* is set very small. For saving the computing time and cost, the program will be terminated early.

## Results

### Simulation experiments

The datasets used in simulation experiments are generated as following:

- Step 1: Supposing the dataset has *n* samples, and the number of the genes is 4000. In these 4000 genes we set 10 disease related genes, and the coefficients of the remaining 3990 genes are set zero.
- Step 2: The correlation coefficient *p* is set 0.3. ${x}_{i}={\gamma }_{i}\sqrt{1-\rho }+{\gamma }_{i0}\sqrt{\rho }$ where ${{\rm{\gamma }}}_{i0},{\gamma }_{i1},\ldots ,{\gamma }_{ip}$ *(i* = 1, *…, n)* are generated independently from standard normal distribution
- Step 3: The sample is generated as: $\mathrm{log}\,\frac{{y}_{i}}{1-{y}_{i}}={\beta }_{0}+{\sum }^{}{x}_{i}\beta +\varepsilon$, where *β*_0_ is the intercept and *ε* is the randomly generated Gauss white noise.
- Step 4: The unlabeled data points are selected randomly, supposing in the dataset there are *n*_*1*_ labeled samples and *n*_2_ unlabeled samples, where *n* = *n*_1_ + *n*_2_. In Group A we suppose *n*_1_ = 100, *n*_2_ = 200; and in Group B *n*_1_ = 150, *n*_2_=300. We recorded the (${y}_{i},{x}_{i},{c}_{i}$), *c*_*i*_ = 0 means the corresponding *y*_*i*_ is unlabeled.

In this paper we compare six different methods: the single logistic model with Lasso, the AL logistic model with Lasso (AL-lo), the self-training logistic model with Lasso (SSL-lo), the logistic model combining with AL and SSL which needs manual labeling (ASSL-lo), the auto logistic model with Lasso combining with AL and SSL without manual labeling and update mechanism (Auto-ASSL(A)), and the logistic model with Lasso combining with AL and SSL without manual labeling but using update mechanism (Auto-ASSL(B)). In AL-lo and ASSL-lo, about 40% unlabeled samples are labeled manually. The classification accuracy of the unlabeled data is used to evaluate the classification performances of different models. The number of selected correct genes (NC), the number of selected genes (NS), *sensitivity* and *specificity* are used to evaluate the gene selection performances of the methods. Supposing true positive (TP) is the number of identified disease related genes, false positive (FP) is the number of selected unrelated genes, false negative (FN) is the number of disease related genes which are missed, and true negative (TN) is the number of the unrelated genes that are abandon by different models. The *sensitivity* and *specificity* can be expressed as:

$$sensitivity=\frac{TP}{TP+FN}$$

$$specificity=\frac{TN}{TN+FP}$$

The gene selection performances of different methods in simulation experiments are shown in Table 1, the results are the average of 100 runs of the program. It is easy to find the *specificity* obtained by single logistic regression model is highest than any other methods, it means it doesn’t select too many unrelated genes. However the lowest *sensitivity* shows single logistic regression model selects the least disease related genes. The AL-lo achieves a closed *specificity* value compared to single logistic model, but it identifies more disease related genes. The SSL-lo selects more disease related genes than single logistic model, and meanwhile many unrelated genes are also selected. Through combining the AL and SSL, the ASSL-lo identifies most disease related genes, but the problem is that it also selects more disease unrelated genes than SSL-lo. Auto-ASSL(A) selects less correct genes compared to ASSL, and the numbers of selected unrelated genes are closed. Compared to the Auto-ASSL(A), the gene selection performance obtained by Auto-ASSL(B) is obviously improved. The *sensitivity* obtained by Auto-ASSL(B) is only less than ASSL-lo but higher than any other methods, and the *specificity* is even more than the ASSL-lo. It shows that the Auto-ASSL(B) can achieve a balance between the *sensitivity* and *specificity*, and it has a strong ability to identify the disease related genes meanwhile eliminates the interference of unrelated genes.

**Table 1:** The gene selection performances of different methods in simulation experiments.

	Group A				Group B			
	NC	NS	sensitivity	specificity	NC	NS	sensitivity	specificity
logistic	3.15	14.05	0.315	0.995	4.82	26.58	0.482	0.989
AL-lo	3.65	17.80	0.365	0.992	5.19	28.16	0.519	0.988
SSL-lo	3.87	23.61	0.387	0.990	5.51	45.40	0.551	0.979
ASSL-lo	5.32	63.90	0.532	0.971	6.74	96.27	0.674	0.955
Auto-ASSL(A)	3.59	57.68	0.359	0.973	5.26	97.39	0.526	0.953
Auto-ASSL(B)	4.17	27.45	0.417	0.988	5.75	53.60	0.575	0.976

**Figure 2:**
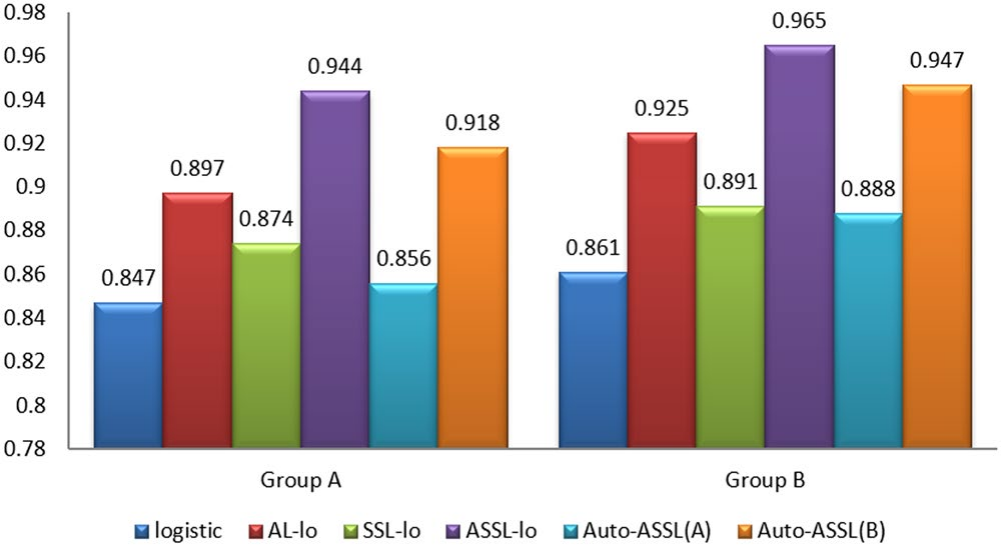
The classification accuracy of different methods in simulation experiments.

**Figure 3:**
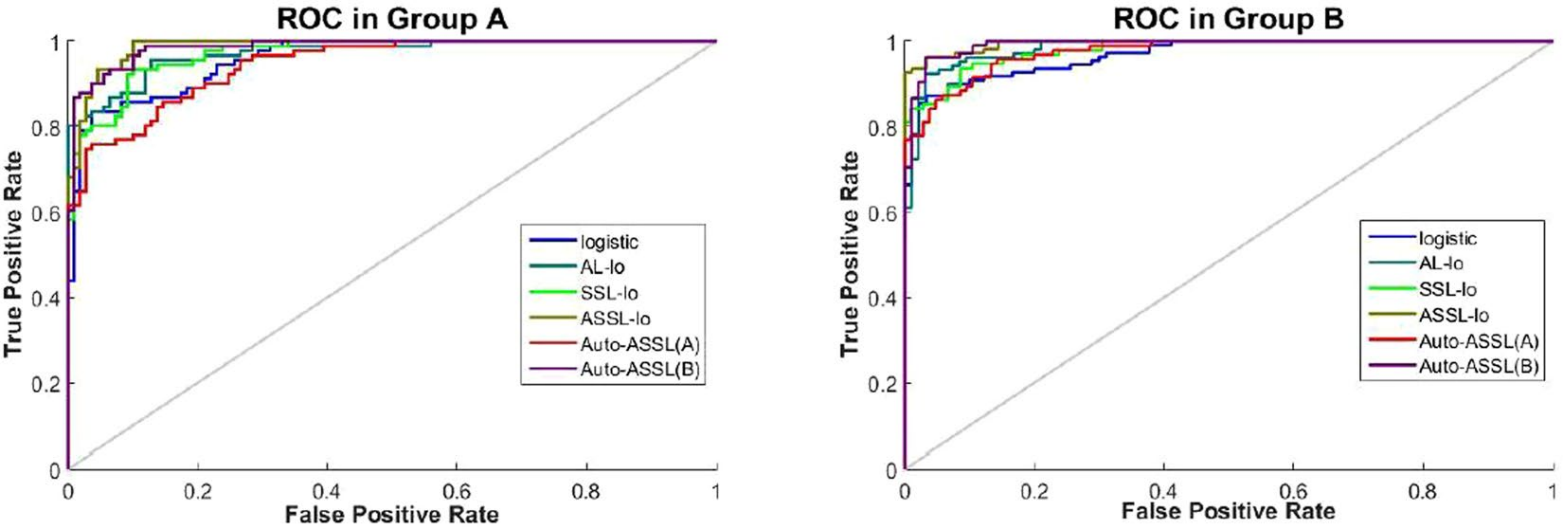
The ROC curves of different methods in simulation experiments.

The values of classification accuracy obtained by different methods in the unlabeled data are shown in Fig. 2. The ROC curves obtained by different methods in one run of the program are shown in Fig. 3. And the AUC values corresponding to the ROC curves are given in Table 2. The ASSL logistic model achieves the best result through combining the AL and SSL, however it needs much time and cost for manual labeling. The performance obtained by Auto-ASSL(A) is even worse than SSL logistic model, this result proves the misclassified uncertain samples have significant bad effect on the classification performance and our update mechanism is very necessary for Auto-ASSL. The results show our method is advanced because it achieves higher accuracy than AL-lo or SSL-lo and only less than ASSL-lo, and meanwhile it doesn’t need any manual labeling.

**Table 2:** The AUC obtained by different methods in simulation experiments.

AUC	logistic	AL-lo	SSL-lo	ASSL-lo	Auto-ASSL(A)	Auto-ASSL(B)
Group A	0.9584	0.9723	0.9709	0.9874	0.9448	0.9810
Group B	0.9682	0.9855	0.9796	0.9943	0.9738	0.9917

Hence the new logistic regression model combing SSL and AL can be seen as a very efficient method because it implements the following functions:

1. It works without any manual intervention. This saves much cost and the results can be quickly obtained.
2. It can achieve accuracy above 90% in disease classification. The experiments show our method can achieve a better accuracy than the AL and SSL logistic regression models.
3. It can identify more disease related genes and at the same time less unrelated genes will be selected. This further saves the researchers’ time and cost.

### Real data experiments

In real data experiments six methods are applied on four real gene expression datasets: Diffuse large B-cell lymphoma (DLBCL) dataset^16^, Prostate cancer dataset^17^, GSE21050^18^ and GSE32603^19^. In these four datasets about 2/3 samples are treated as the unlabeled samples for evaluating the classification accuracy of unlabeled samples. The labeled samples and unlabeled samples are randomly selected in every runs of the program. More details of the datasets used in the experiments are shown in Table 3.

**Table 3:** Details of real datasets used in the experiments.

Dataset	Number of genes	Number of samples	Number of labeled samples	Disease types
DLBCL	2648	77	26	diffuse large b-cell lymphoma
Prostate	2135	102	34	prostate cancer
GSE21050	54613	310	103	soft tissue sarcomas
GSE32603	13200	231	77	breast cancer

**Figure 4:**
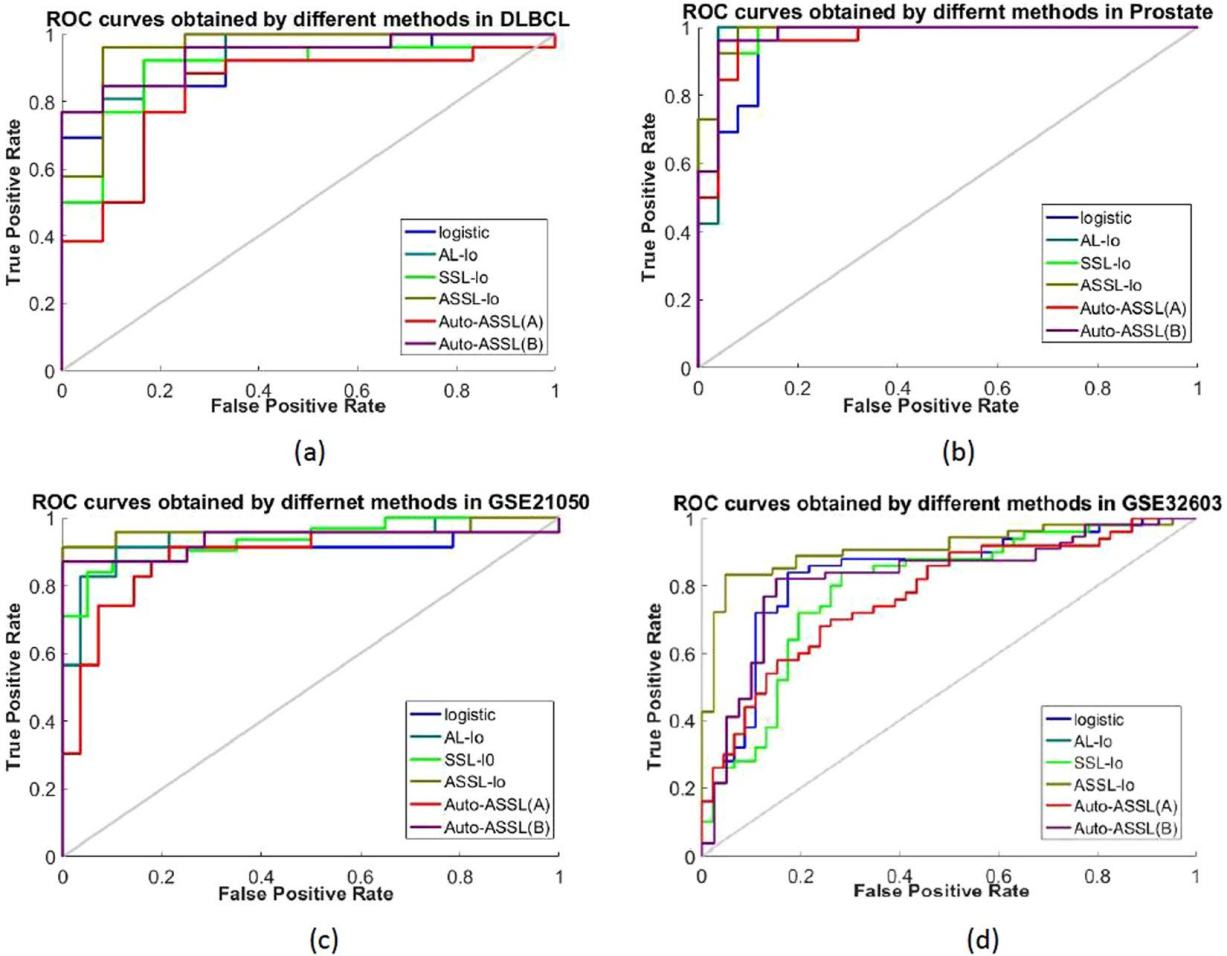
ROC curves obtained by different methods in real datasets (**a**) DLBCL (**b**) Prostate (**c**) GSE21050 (**d**) GSE32603.

The values of classification accuracy obtained by different methods in real datasets are shown in Table 4. The ROC curves obtained by different methods in one run of the program in different datasets are shown in Fig. 4, and the corresponding AUC are shown in Table 5. The SSL-lo performs better than single logistic and Auto-ASSL(A), but worse than the other three methods. It is obviously that the accuracy of ASSL logistic model is highest. The Auto-ASSL(A) does not perform well because the misclassified samples affect the accuracy. The classification accuracy obtained by Auto-ASSL(B) is better than any other methods except ASSL which proves that the update pseudo-labeled samples mechanism is a very important improvement for the model.

**Table 4:** The classification accuracy obtained by different methods in the real datasets.

Method	DLBCL	Prostate	GSE21050	GSE32603
logistic	77.94%	86.54%	79.01%	69.69%
AL-lo	83.15%	91.53%	84.43%	73.68%
SSL-lo	81.82%	88.97%	80.92%	70.57%
ASSL-lo	87.14%	94.42%	89.34%	80.63%
Auto-ASSL(A)	80.67%	88.55%	78.33%	68.48%
Auto-ASSL(B)	85.62%	93.36%	86.37%	76.46%

**Table 5:** The AUC obtained by different methods in the real datasets.

Method	DLBCL	Prostate	GSE21050	GSE32603
logistic	0.9199	0.9569	0.8975	0.7557
AL-lo	0.9295	0.9749	0.9394	0.8328
SSL-lo	0.8942	0.9708	0.9232	0.7962
ASSL-lo	0.9583	0.9862	0.9596	0.9023
Auto-ASSL(A)	0.8333	0.9646	0.8835	0.7757
Auto-ASSL(B)	0.9391	0.9785	0.9432	0.8390

**Figure 5:**
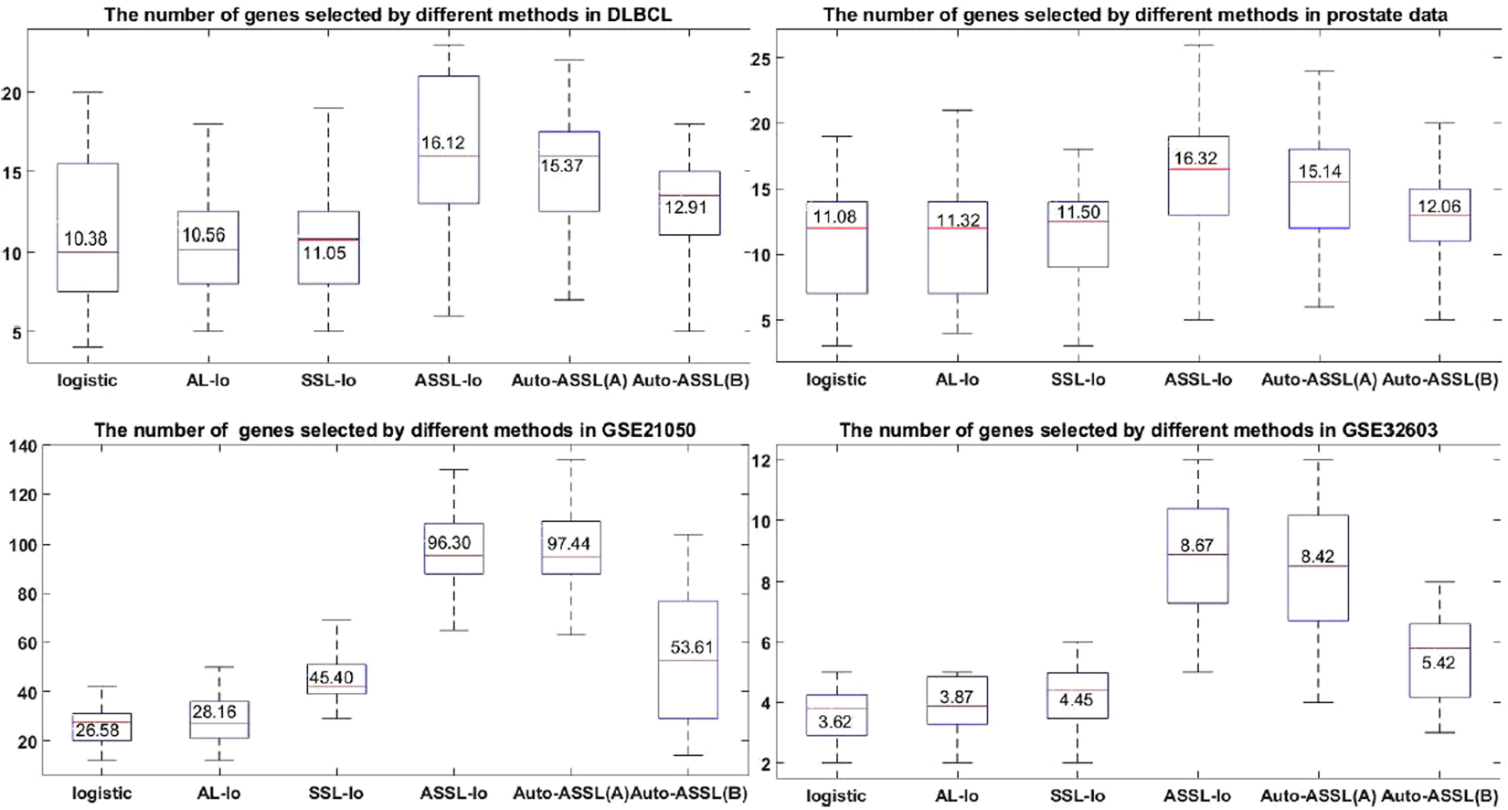
The number of genes selected by different methods in real datasets.

The numbers of genes selected by different methods in real dataset are shown in Fig. 5. It is obvious that the single logistic method selects least genes. The numbers of selected genes obtained by ASSL and Auto-ASSL(A) are far more than other methods. Our method selects more genes than AL-lo and SSL-lo, but less than ASSL-lo.

In order to further assess the correctness of the selected genes by different methods, the top-10 ranked genes selected by different methods in real datasets are listed in Tables 6–9, Table 9 is partly blank because the methods didn’t select so many genes. The genes in italic in the tables such as *SELENOP, HPN, MTHFD2* and *ROR2* are the ones which are selected by all the methods in the same datasets. The *SELENOP* in *DLBCL* can be seen as an extracellular antioxidant, and it may be potential non-invasive diagnostic markers for cancer. Some researches show that selenium could be seen as an anticancer therapy by affecting *SELENOP*^20^. The research has proved that expression of the encoded protein of *HPN* is related to the growth and progression of cancers, particularly prostate cancer. It may be associated with susceptibility to prostate cancer^21^. The *MTHFD2* in *GSE21050* is seen as a prognostic factor and a potential therapeutic target for future cancer treatments.^22^. The *ROR2* in *GSE32603* is reported that it can significantly reduce cell proliferation and induced apoptosis^23^.

**Table 6:** The genes selected by different methods in DLBCL.

	logistic	AL-lo	SSL-lo	ASSL-lo	Auto-ASSL(A)	Auto-ASSL(B)
1	*SELENOP*	*SELENOP*	*SELENOP*	*SELENOP*	*SELENOP*	**MDM4**
2	KIF2C	MT2A	PURA	CD34	GPR18	*SELENOP*
3	MT2A	MIF	MT2A	TXNIP	ESD	MIF
4	MORC3	*GLIPR1*	*GLIPR1*	MT2A	*GLIPR1*	MORC3
5	TLE4	SELL	TLE4	PURA	SELL	TLE4
6	SELL	BMI1	MIF	TRIB2	MYCLP1	SELL
7	N4BP2L1	IFITM2	N4BP2L1	GAPDH	TRIM23	N4BP2L1
8	*GLIPR1*	GAPDH	SELL	MYCLP1	TLE4	*GLIPR1*
9	EFNA3	CCL21	CCL21	*GLIPR1*	KIF2C	CCL21
10	MYCLP1	SMAD6	ESD	MIF	GAPDH	MT2A

**Table 7:** The genes selected by different methods in Prostate.

	logistic	AL-lo	SSL-lo	ASSL-lo	Auto-ASSL(A)	Auto-ASSL(B)
1	*HPN*	*TP63*	*TP63*	*TP63*	PTGDS	*TP63*
2	*TP63*	XBP1	XBP1	XBP1	*HPN*	*HPN*
3	MYOF	NELL2	*HPN*	*HPN*	NELL2	MYOF
4	XBP1	TGFB3	PTGDS	NELL2	RRAD	XBP1
5	PTGDS	*HPN*	NELL2	RBM3	HSBP1	**JUNB**
6	NELL2	ATP5ME	MYOF	PTGDS	MYOF	NELL2
7	SERPINA3	TRIM29	ATP5ME	SDC1	*TP63*	SERPINA3
8	RBM3	MYOF	SERPINA3	CFD	PDLIM5	**TIPARP**
9	TGFB3	RBM3	TGFB3	ATP5ME	ATP5ME	TGFB3
10	TRIM29	SERPINA3	TRIM29	HSBP1	SERPINA3	TRIM29

**Table 8:** The genes selected by different methods in GSE21050.

	logistic	AL-lo	SSL-lo	ASSL-lo	Auto-ASSL(A)	Auto-ASSL(B)
1	C15orf41	*SNAPC1*	*SNAPC1*	FADS1	*MTHFD2*	*MTHFD2*
2	*SNAPC1*	SNORD35B	C8orf82	SNORD35B	ADD3	SNORD35B
3	C8orf82	*MTHFD2*	*MTHFD2*	IFT43	*SNAPC1*	*SNAPC1*
4	*MTHFD2*	NFATC2IP	SLC1A4	C8orf82	SNORD35B	ADD3
5	LPAR1	C8orf82	PML	CDC42EP3	FHL2	C8orf82
6	AKT2	NUP155	PLD1	*MTHFD2*	PCDH18	XPO6
7	XPO6	XPO6	WDHD1	DCN	NFATC2IP	ATP6V1D
8	SLC1A4	IFT43	AKT2	*SNAPC1*	YEATS2	IFT43
9	PLD1	PCDH18	RPL13A	XPO6	LIMK2	NUP155
10	SNORD35B	WDHD1	NFATC2IP	ADD3	SMAD4	**ENO2**

**Table 9:** The genes selected by different methods in GSE32603.

	logistic	AL-lo	SSL-lo	ASSL-lo	Auto-ASSL(A)	Auto-ASSL(B)
1	*ROR2*	GRB2	LOC642236	*ROR2*	*ROR2*	*EMB*
2	LOC642236	MS4A1	*ROR2*	*EMB*	*EMB*	*ROR2*
3	GRB2	*EMB*	GRB2	ZSCAN9	GRB2	GRB2
4	*EMB*	*ROR2*	*EMB*	CDKN1B	TMEM242	TMEM242
5			MAST1	UBE2W	UFC1	HPSE
6				C2orf70	EPHB1	**TPD52L2**
7				TAF8	SUPT20H	
8				MTSS1	ARL2BP	
9				PRDM4	STK3	

On the other hand, our method also identified some special genes which other methods did not select.

These genes are shown in bold in the Tables 6–9. The *MDM4* in *DLBCL* plays a very important role in the proliferation of the cancer cells, and it is crucial for the establishment and progression of tumors^24^. *JUNB* plays a specific role in cancer cell proliferation, survival and drug resistance^25^. Single nucleotide polymorphism of *TIPARP* in *Prostate* has been proved to be related with cancer^26^. In^27^ *ENO2* is reported to be a risk factor for bone metastases in cancer. The *TPD52L2* in *GSE32603* encodes a member of the tumor protein D52-like family, and contributes to proliferation of cancer cells^28^. These genes mentioned in the literatures demonstrate that our new logistic regression model has a strong ability in gene selection.

## Conclusion

In this paper we have designed a novel method which does not require significant engineering overhead to use and meanwhile achieves satisfying results by utilizing the unlabeled gene expression samples in disease classification. The novel logistic regression model is designed based on the complementarity of semi-supervised learning and active learning. In addition to that an update pseudo-labeled samples mechanism is embedded in this method to reduce the false pseudo-labeled samples. In conclusion, our method can achieve more accuracy results compared widely used SSL and AL logistic models, and it also has a good performance in identifying the disease related genes. In addition to that, this model can work without any manual labeling for saving much time and cost. We believe it will be an efficient tool to make contributions for disease classification and gene selection because of its high reliability and stability against noise and outliers.

## References

[1] King G, Zeng L (2001). Logistic regression in rare events data. Political analysis.

[2] Gunn SR (1998). Support vector machines for classification and regression. ISIS technical report..

[3] Zhu X. Semi-supervised learning literature survey. *Computer Science*. 2–4 (2006).

[4] Fu, Y., Zhu, X. & Li, B. A survey on instance selection for active learning. *Knowledge and information systems*. 1–35 (2013).

[5] Lewis, D. D. & Catlett, J. Heterogeneous uncertainty sampling for supervised learning. *Proceedings of the eleventh international conference on machine learning*. 148–156 (1994).

[6] Settles, B. Active learning literature survey. *University of Wisconsin, Madison*. 55–66 (2010).

[7] Kasabov, N. & Pang, S. Transductive support vector machines and applications in bioinformatics for promoter recognition. *Neural networks and signal processing*. 1–6 (2003).

[8] Goldberg, A. B., Zhu, X. & Wright, S. Dissimilarity in graph-based semi-supervised classification. *Artificial Intelligence and Statistics*. 155–162 (2007).

[9] Nigam, K. & Ghani, R., Analyzing the effectiveness and applicability of co-training. *Proceedings of the ninth international conference on Information and knowledge management*. 86–93 (2000).

[10] Rosenberg, C., Hebert, M. & Schneiderman, H. Semi-supervised self-training of object detection models (2005).

[11] Li YF, Zhou ZH (2015). Towards making unlabeled data never hurt. IEEE Transactions on Pattern Analysis and Machine Intelligence..

[12] Song M, Yu H, Han WS (2011). Combining active learning and semi-supervised learning techniques to extract protein interaction sentences. BMC bioinformatics..

[13] Zhu, X., Lafferty, J., Ghahramani, Z. Combining active learning and semi-supervised learning using gaussian fields and harmonic functions. *ICML 2003 workshop on the continuum from labeled to unlabeled data in machine learning and data mining*. **3** (2003).

[14] Leng Y, Xu X, Qi G (2013). Combining active learning and semi-supervised learning to construct SVM classifier. Knowledge-Based Systems..

[15] Lin L (2018). Active self-paced learning for cost-effective and progressive face identification. IEEE transactions on pattern analysis and machine intelligence..

[16] Shipp MA (2002). Diffuse large B-cell lymphoma outcome prediction by gene-expression profiling and supervised machine learning. Nature medicine..

[17] Singh D (2002). Gene expression correlates of clinical prostate cancer behavior. Cancer cell..

[18] Chibon F (2010). Validated prediction of clinical outcome in sarcomas and multiple types of cancer on the basis of a gene expression signature related to genome complexity. Nature medicine..

[19] Magbanua MJM (2015). Serial expression analysis of breast tumors during neoadjuvant chemotherapy reveals changes in cell cycle and immune pathways associated with recurrence and response. Breast Cancer Research..

[20] Tarek, M. *et al*. Role of microRNA-7 and selenoprotein P in hepatocellular carcinoma. *Tumor Biology*. **39** (2017). 10.1177/1010428317698372 28459371

[21] Kim HJ (2012). Variants in the HEPSIN gene are associated with susceptibility to prostate cancer. Prostate cancer and prostatic diseases..

[22] Liu F (2014). Increased MTHFD2 expression is associated with poor prognosis in breast cancer. Tumor Biology.

[23] Yang (2017). Ror2, a Developmentally Regulated Kinase, Is Associated With Tumor Growth, Apoptosis, Migration, and Invasion in Renal Cell Carcinoma. Oncology Research Featuring Preclinical and Clinical Cancer Therapeutics.

[24] Miranda (2017). MDM4 is a rational target for treating breast cancers with mutant p53. The Journal of pathology.

[25] Fan F (2017). The AP-1 transcription factor JunB is essential for multiple myeloma cell proliferation and drug resistance in the bone marrow microenvironment. Leukemia.

[26] Goode (2010). A genome-wide association study identifies susceptibility loci for ovarian cancer at 2q31 and 8q24. Nature genetics..

[27] Zhou (2017). Neuron-specific enolase, histopathological types, and age as risk factors for bone metastases in lung cancer. Tumor Biology.

[28] Zhou (2013). hABCF3, a TPD52L2 interacting partner, enhances the proliferation of human liver cancer cell lines *in vitro*. Molecular biology reports.

